# A pro‐inflammatory cytokine signature characterises thrombotic antiphospholipid syndrome despite stable anticoagulation

**DOI:** 10.1111/bjh.70816

**Published:** 2026-09-09

**Authors:** Catherine B. Ducker, Megan V. Preece, Jeremy Schofield, Charis Pericleous, Mike Laffan, Simon Abrams, Deepa J. Arachchillage

**Affiliations:** ^1^ Centre for Haematology, Department of Immunology and Inflammation Imperial College London London UK; ^2^ Department of Clinical Infection, Microbiology and Immunology University of Liverpool Liverpool UK; ^3^ Roald Dahl Haemostasis and Thrombosis Centre Liverpool University Hospitals NHS Foundation Trust Liverpool UK; ^4^ National Heart and Lung Institute Imperial College London London UK; ^5^ Department of Haematology Imperial College Healthcare NHS Trust London UK

**Keywords:** antiphospholipid syndrome, cytokines, inflammation, thrombosis

## Abstract

Inflammation contributes to primary antiphospholipid syndrome (PAPS) pathogenesis, but cytokine profiles and associated thrombotic phenotypes remain incompletely defined. A panel of 20 cytokines, chemokines and endothelial activation markers was analysed in 37 PAPS patients on stable anticoagulation and 12 healthy controls (HC). We compared concentrations between groups and performed unsupervised hierarchical clustering and principal component analysis (PCA) to identify inflammatory subgroups. Compared with HC, patients with PAPS exhibited significantly elevated levels of 14/20 analytes: granulocyte‐macrophage colony‐stimulating factor (GM‐CSF), interferon (IFN)‐γ, interleukin (IL)‐1α, IL‐1β, IL‐6, IL‐10, IL‐12p70, IL‐13, IL‐17a, tumour necrosis factor‐α (TNF‐α), intercellular adhesion molecule‐1 (ICAM‐1), P‐selectin and macrophage inflammatory protein‐1β (MIP1β) (all *p* < 0.05). Unsupervised clustering identified three inflammatory antiphospholipid syndrome (APS) subgroups distinct from HC. A core cytokine signature comprising GM‐CSF, IL‐6, IL‐10, IL‐12p70, IL‐13 and TNF‐α was elevated across all APS clusters. One cluster, enriched for arterial thrombosis, demonstrated a more pronounced inflammatory phenotype, with increased IFN‐α. Comparison of phenotypes showed significantly higher concentrations of GM‐CSF, IFN‐α, IL‐1α, IL‐4, IL‐6, IL‐10, IL‐17a and TNF‐α in arterial versus venous thrombosis. Our findings demonstrate widespread cytokine dysregulation and distinct inflammatory subgroups in PAPS, with arterial thrombosis showing stronger inflammation than venous thrombosis, supporting divergent thrombo‐inflammatory mechanisms and potential precision immunomodulatory therapeutic targets.

## Graphical abstract

Primary antiphospholipid syndrome comprises distinct inflammatory subgroups defined by cytokine profiling. A shared pro‐inflammatory signature is present across all patients, whereas arterial thrombosis demonstrates enhanced inflammatory activation compared with venous thrombosis, suggesting divergent pathogenic mechanisms and opportunities for precision immunomodulatory therapy.

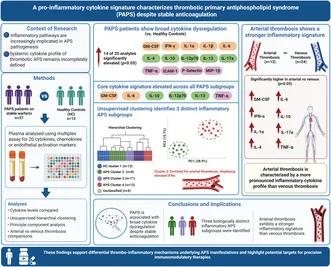

Antiphospholipid syndrome (APS) is an acquired autoimmune disorder characterised by recurrent arterial and/or venous thrombosis and pregnancy morbidity in the persistent presence of antiphospholipid antibodies (aPL).^1^ Although the clinical manifestations of thrombotic APS are well defined, the immunopathogenical mechanisms linking aPL to a sustained prothrombotic state remain incompletely understood. Growing evidence indicates that APS extends beyond a coagulation disorder, representing a thrombo‐inflammatory disease driven by immune activation and endothelial dysfunction.^2^

Cytokines are key mediators of immune and inflammatory responses, implicated in the regulation of coagulation, platelet activation and vascular homeostasis.^3^ Experimental and clinical studies have suggested that aPL can induce cytokine release from monocytes, endothelial cells and platelets, thereby amplifying inflammatory signalling pathways that promote thrombosis.^4^, ^5^ However, data on systemic cytokine profiles in patients with thrombotic primary antiphospholipid syndrome (PAPS) remain limited and heterogeneous.^6^, ^7^

Although inflammatory cytokine abnormalities have been described in APS, whether distinct cytokine‐defined inflammatory endotypes exist within thrombotic APS and whether these differ according to thrombotic phenotype remain unknown. Therefore, we investigated circulating cytokine profiles in patients with primary thrombotic APS, without associated autoimmune diseases, compared with healthy controls (HC). Using multiplex cytokine analysis and unsupervised clustering approaches, we characterised the inflammatory cytokine dysregulation associated with APS‐related thrombosis, identified distinct cytokine‐defined inflammatory endotypes and their relationship to thrombotic phenotype, exploring potential biomarkers and therapeutic targets within the thrombo‐inflammatory axis.

## PATIENTS AND METHODS

Patients with a confirmed diagnosis of primary thrombotic APS based on both revised Sapporo (‘Sydney’) criteria and American College of Rheumatology (ACR)/European League Against Rheumatism (EULAR) classification, attending a dedicated APS clinic at Imperial College Healthcare National Health Service (NHS) Trust, London, United Kingdom, between August 2022 and January 2023, who provided informed written consent were included in this study. Inclusion criteria comprised (1): single thrombotic event, either venous or arterial thrombosis at least 3 months prior to the study, and (2) persistent single, double or triple positivity for aPL detected 12 weeks apart. Only patients with immunoglobulin G (IgG) or immunoglobulin M (IgM) anti‐cardiolipin (aCL) and/or anti‐β_2_glycoprotein 1 (αβ_2_GPI) antibody levels of ≥40GPL (IgG phospholipid) or MPL (IgM phospholipid) units measured by Enzyme‐linked Immunosorbent Assay (ELISA) were considered positive. Lupus anticoagulant (LA) was tested as per current guidelines.^1^, ^8^ To avoid the possible confounding effect of anti‐thrombotic treatment on cytokine levels, only patients on stable anticoagulation with warfarin (at least 3 months on warfarin with the time of international normalised ratio (INR) in the therapeutic range ≥ 70%) with a target INR of 2.5 (2.0–3.0) were included. Patients with obstetric APS or both obstetric and thrombotic APS, those with other autoimmune diseases, ischemic heart disease/coronary events or on anti‐platelet treatment were excluded. The study was approved by the Human Research Authority and Health and Care Research Wales (reference:22/PR/145). Blood samples were obtained from patients at routine clinic visits. Citrated blood samples were double centrifuged at 2000*g* for 15 min, plasma separated and stored at −80°C, until analysis.

A total of 37 primary APS patients met the inclusion criteria. Blood samples from 12 HC were collected, processed and stored in the same way.

### Measurement of cytokines

Twenty cytokines, chemokines and cell adhesion activation markers (cytokines: granulocyte‐macrophage colony‐stimulating factor [GM‐CSF], interferon [IFN]‐α, IFN‐γ, interleukin [IL]‐1α, IL‐1β, IL‐4, IL‐6, IL‐8, IL‐10, IL‐12p70, IL‐13, IL‐17a and tumour necrosis factor‐α [TNF‐α]; chemokines: C–X–C motif chemokine 10 [CXCL10] also known as IFN‐γ‐induced protein [IP‐10], monocyte chemoattractant protein‐1 [MCP‐1], macrophage inflammatory protein‐1 α [MIP1α], and MIP1β; adhesion activation markers: intercellular adhesion molecule‐1 [ICAM‐1], E‐selectin and P‐selectin) were measured by MultiPlex (Thermo Fisher Scientific) in plasma of patients or HC. The assay was undertaken according to the manufacturer's instructions, using the Bio‐Plex 100 instrument.

### Data analysis

Analysis was performed with GraphPad Prism v10.4.1 and R v4.4.3.^9^ R packages included ggplot2,^10^ reshape2^11^ and amap.^12^

Hierarchical clustering and heatmap analysis were performed by initially transforming the cytokine concentrations by using log(*x* + 1). Log normalised data was then z‐scaled. Hierarchical clustering using the amap package was undertaken using the Spearman method. Samples and cytokines were re‐ordered according to their position in the dendrogram and plotted using ggplot2. Clusters were identified by dendrogram grouping of ordered samples. Principal component analysis (PCA) was undertaken using base R and the function prcomp().

Comparison of cytokine titres between APS and HC, and between venous and arterial thrombosis was undertaken in GraphPad Prism, using unpaired *t*‐tests with and without Welch's correction. The individual cytokine analyses represent exploratory biomarker rather than confirmatory hypothesis testing. For this reason, unadjusted *p* values are presented to minimise the risk of overlooking potentially relevant biological signals in this relatively small pilot cohort.

Analysis of cytokine differences between clusters used base R, Wilcoxon test, with the resulting *p*‐value multiplied by the number of cytokines tested to give *p.adj*. Values between clusters were reported as significant if both the *p.adj* < 0.05 and the log(2) fold‐change was greater than ±1.

## RESULTS

There was no difference in the age, biological sex or body mass index (BMI) between patients and controls. Of 37 APS patients included in the study, 24 had venous thrombosis (12 deep vein thrombosis [DVT], eight pulmonary embolism [PE], four with DVT and PE). The remaining 13 had arterial thrombosis (11 stroke, one with abdominal aorta thrombosis and one with left renal artery thrombosis). Baseline characteristics are presented in Table 1. There was no difference in age, biological sex or BMI, aPL profile or comorbidities or treatments between patients with venous versus arterial thrombosis (Table 1). None of the single or double positive aPL patients, but 6/8 (75%) aPL patients with venous thrombosis and 5/6 (83%) triple positive with arterial thrombosis were receiving hydroxychloroquine (HCQ).

**TABLE 1:** Baseline characteristics, aPL profile, comorbidities and APS specific treatment for patients with venous versus arterial thrombosis.

Characteristics	Venous thrombosis (*N* = 24)	Arterial thrombosis (*N* = 13)	*p*‐value
General characteristics			
Age—median (range) years	56 (22–64)	54 (23–64)	0.65
Biological sex (male/female)	7/18	7/6	0.16
BMI—median (range) kg/m^2^	24.2 (21.0–34.0)	26.1 (21.0–34.0)	0.37
Time since thrombotic event (months)	23 (9–36)	22 (8–37)	0.52
aPL profile			
Triple positive	8 (33.3%)	6 (46.1%)	0.48
Double positive (αβ_2_GPI and anti‐cardiolipin)	9 (37.5%)	5 (38.5%)	0.99
Single positive	7^a^ (29.2%)	2^b^ (15.4%)	0.45
Comorbidities			
Smoking	5 (20.8%)	3 (23.1%)	0.99
Hypertension	8 (33.4%)	2 (15.4%)	0.44
Type 2 diabetes mellitus	5 (20.8%)	3 (23.1%)	0.99
Dyslipidaemia	6 (25%)	4 (30.8%)	0.70
APS treatment			
Warfarin	24 (100%)	13 (100%)	0.99
Statin	6 (25%)	4 (30.8%)	0.70
Hydroxychloroquine	6 (25%)	5 (38.5%)	0.45

Abbreviations: aPL, antiphospholipid antibodies; APS, antiphospholipid syndrome; BMI, body mass index.

^a^ 5/7 patients are lupus anticoagulant positive.

^b^ 2 patients are lupus anticoagulant positive.

### Thrombotic APS patients exhibit pro‐inflammatory cytokine profiles

Compared to controls, APS patients showed significantly elevated levels of 14/20 cytokines measured: GM‐CSF, ICAM‐1, IFN‐γ, IL‐1α, IL‐1β, IL‐4, IL‐6, IL‐10, IL‐12p70, IL‐13, IL‐17a, MIP1β, P‐selectin and TNF‐α (Figure 1). IL‐8 was only elevated in a few samples. Levels of IFN‐α, IP‐10, MCP‐1, MIP1α and E‐selectin did not significantly differ between the two groups (Figure S1).

**FIGURE 1:**
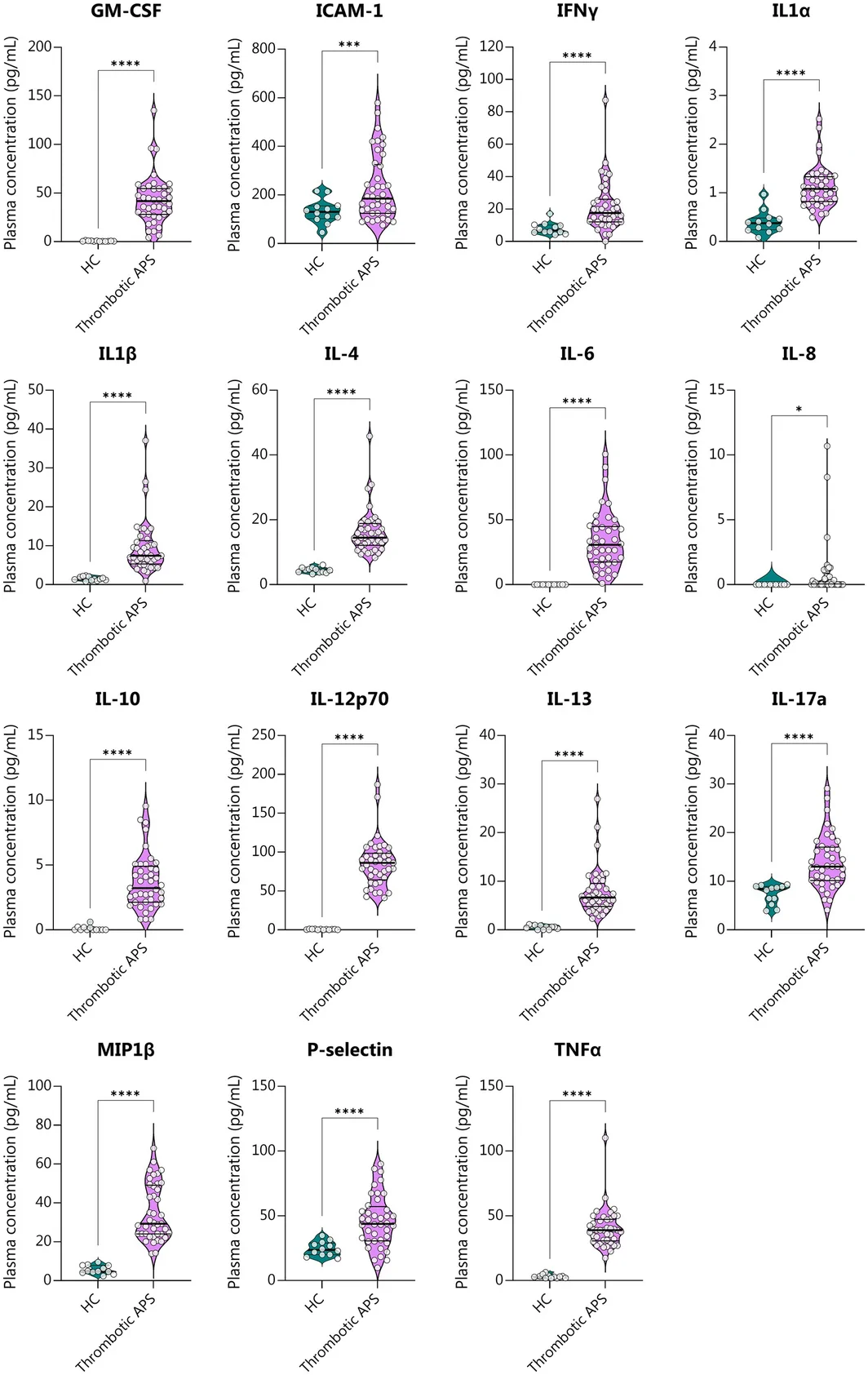
Cytokine plasma concentrations are elevated in thrombotic APS patients compared to healthy controls. Cytokine concentrations were measured in plasma of thrombotic APS patients (*n* = 37) and healthy controls (HC, *n* = 12) by Multiplex assay. The median is identified with a thick black line, and quartiles with thin black lines. Statistical significance was tested for using an unpaired *t* test with Welch's correction. *p < 0.05, **p < 0.01, ***p < 0.005, ****p < 0.0001.

### Hierarchical clustering identifies four distinct patient clusters according to cytokine profile

PCA analysis identified that neither age, BMI, nor biological sex were associated with variation in the data, and thus correction was not undertaken for these parameters (Figure S2). Hierarchical clustering was subsequently performed by cytokine expression and by patient sample (Figure 2). One sample was excluded from hierarchical clustering due to missing cytokine values. Four distinct clusters (clusters 1–4) of samples were identified. Detailed clinical, laboratory and treatment phenotypes of different clusters are summarised in Table 2. Cluster 1 consists of HC, while clusters 2–4 separate thrombotic APS patients into three groups. Clusters 2 and 4 primarily consist of venous thrombosis samples (7/9 and 10/13, respectively), while cluster 3 is enriched with patients with arterial thrombosis (7/11). Three patients did not fit into a large enough cluster for downstream analysis. These patients were a 22‐year‐old man with double positive aPL (IgG aCL and αβ_2_GPI) associated arterial thrombosis, a 54‐year woman with double positive aPL (IgG aCL and αβ_2_GPI) associated venous thrombosis and a 57‐year woman with single positive (LA) associated venous thrombosis.

**FIGURE 2:**
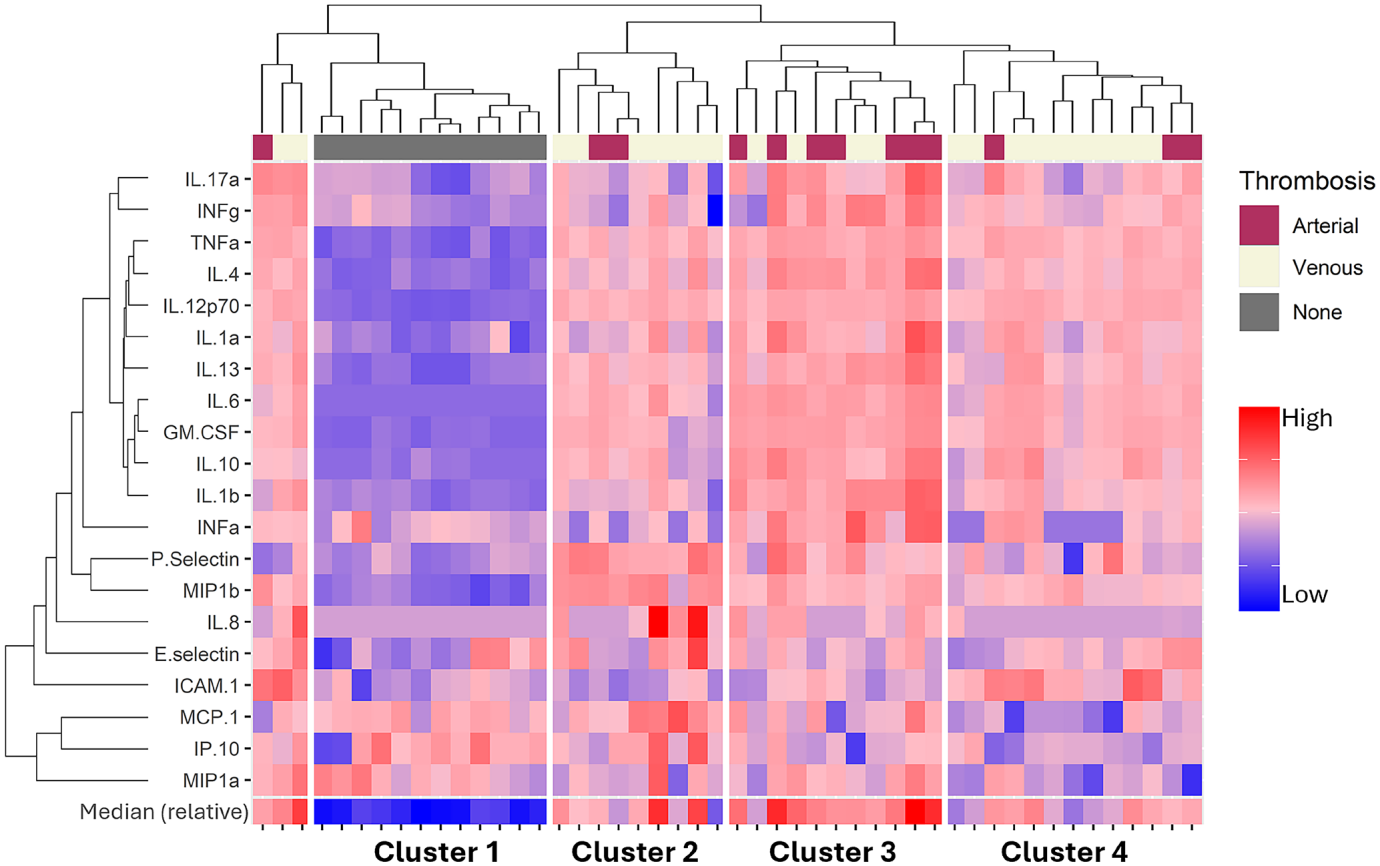
Clustering of plasma samples by cytokine expression. Raw plasma cytokine concentrations were transformed by log(*x* + 1) before normalisation by z‐scaling and hierarchical clustering. Z‐score was plotted as a Heatmap. The x‐axis dendrogram indicates four distinct patient‐sample clusters, with a fifth consisting of too few samples to be regarded a cluster. The thrombosis event type (arterial [*n* = 13], venous [*n* = 24], or none [healthy controls, *n* = 12]) is indicated at the top of the heatmap. For each sample, the median value of all cytokine z‐scores was identified and plotted at the bottom of the heatmap, where colour is relative to the other median values.

**TABLE 2:** Baseline characteristics, aPL profile, comorbidities and APS specific treatment in patients with thrombotic APS versus healthy controls identified in different clusters.

	Cluster 1 (HC) *n* = 12	Cluster 2 *n* = 9	Cluster 3 *n* = 11	Cluster 4 *n* = 13
Age—median (range) years	51 (24–66)	50 (24–62)	47 (22–66)	53 (22–69)
Biological sex (male/female)	4/8	3/6	6/5	4/9
BMI—median (range) kg/m^2^	23.5 (21.0–29.4)	24.1 (21.0–34.0)	23.3 (21.1–34.2)	24.1 (21.1–30.5)
Venous thrombosis	N/A	7	4	10
Arterial thrombosis	N/A	2	7	3
Time since thrombotic event (months)	N/A	22 (8–36)	23 (8–37)	21 (9–35)
aPL profile				
Triple positive	N/A	6	4	3
Double positive	N/A	1	5	7
Single positive	N/A	2^a^	2^a^	3^a^
Comorbidities				
Smoking	1	1	2	5
Hypertension	1	2	3	5
Type 2 Diabetes mellitus	0	2	2	4
Dyslipidaemia	2	2	3	5
APS treatment				
Warfarin	0	9	11	13
Statin	0	2	3	3
Hydroxychloroquine	0	5	4	2

^a^ Two patients with single positive in cluster 2 and 3 are positive for lupus anticoagulant (LA) and 2/3 patients with single positive aPL in cluster 4 are LA positive.

When comparing cytokine concentrations between cluster 1 (HC) and the three APS clusters (clusters 2–4), GM‐CSF, IL‐6, IL‐10, IL‐12p70, IL‐13 and TNF‐α are significantly increased by at least 1 log(2)‐fold in each of clusters 2–4, identifying a group of cytokines consistently higher in thrombotic APS. In addition, MIP1β is significantly higher exclusively in cluster 2, IL‐8 is significantly higher in clusters 2 and 3, IFN‐α in cluster 3, and both IL‐1α and IL‐1β are higher in both clusters 3 and 4 compared to HC (Figure 3).

**FIGURE 3:**
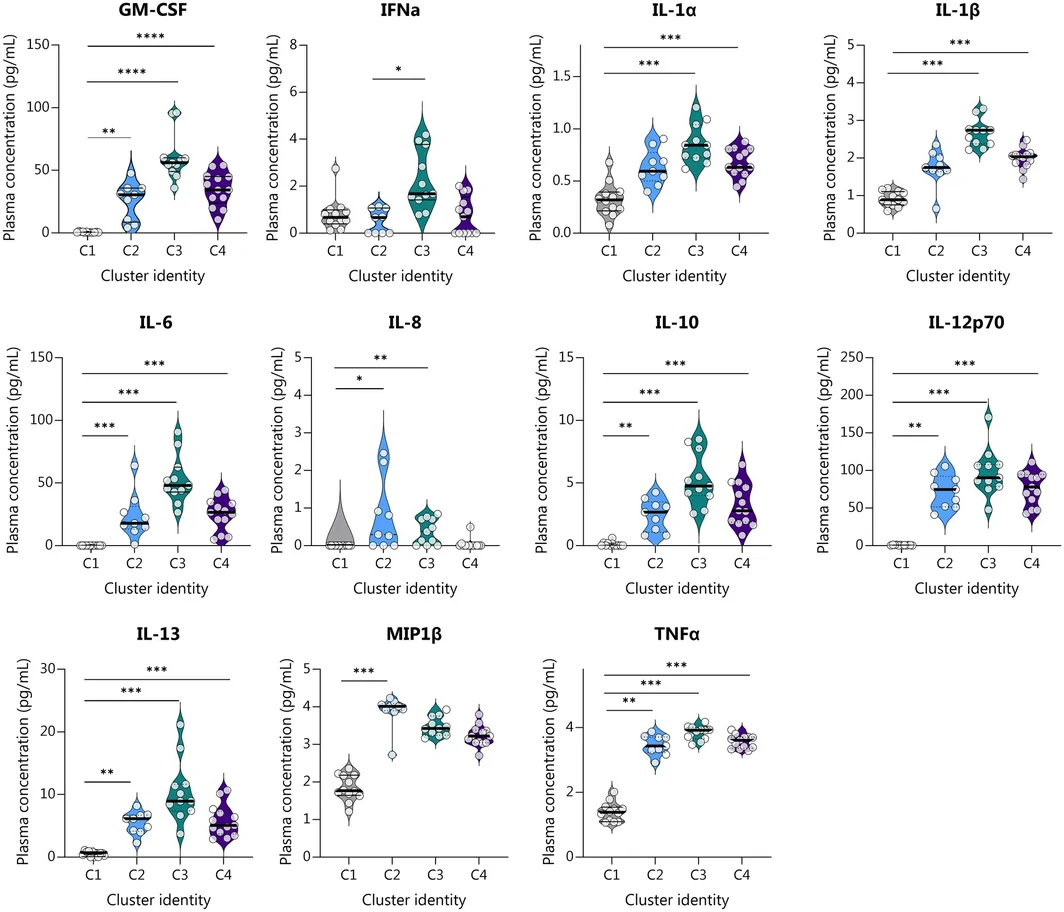
Cytokine concentration by cluster. Plasma cytokine concentrations were compared between cluster 1 (C1 [healthy control], *n* = 12), cluster 2 (C2, *n* = 9), cluster 3 (C3, *n* = 10) and cluster 4 (C4, *n* = 13). Cytokines significantly different between each cluster were identified using wilcox. test(), with *p*‐value adjustment for multiple comparisons across cytokines. Violin plots show the median as a thick black line and quartiles with thin black lines. *p < 0.05, **p < 0.01, ***p < 0.005, ****p < 0.0001.

Comparing APS clusters 2–4, a stronger inflammatory profile is observed in cluster 3 compared to the other thrombotic APS clusters, as visualised by the median cytokine concentration depicted in the heatmap (Figure 2). Specifically, IFN‐α is increased in cluster 3 compared to cluster 2 (1.6 log(2)‐fold, *p.adj* < 0.05, Figure 3), the only cytokine with more than ±1 log(2) fold‐change between clusters.

While hierarchical clustering identifies arterial thrombosis enrichment in the more pro‐inflammatory cluster 3, PCA analysis of overall cytokine profile does not completely separate plasma samples from arterial and venous thrombosis (Figure 4A). This is unsurprising considering the core group of cytokines that is increased in all thrombotic APS, and that venous and arterial thrombosis are subgroups of the same disorder. Despite incomplete separation across PC1 and PC2 axes, a statistically significant difference in the association with the PC1 axis is observed between venous and arterial groups (*p* < 0.05). Surprisingly, aPL status does not appear to be associated with the variation observed (Figure 4B), *p* = 0.3 In addition, aPL titres; complement; HCQ use and vitamin D insufficiency (<50 nmol/L) are not associated with variation across PC1 (Figure S3). Furthermore, cytokine concentrations were compared between HCQ‐treated and non‐HCQ‐treated patients, and no significant differences were observed except for a small increase in MIP1β (Figure S4).

**FIGURE 4:**
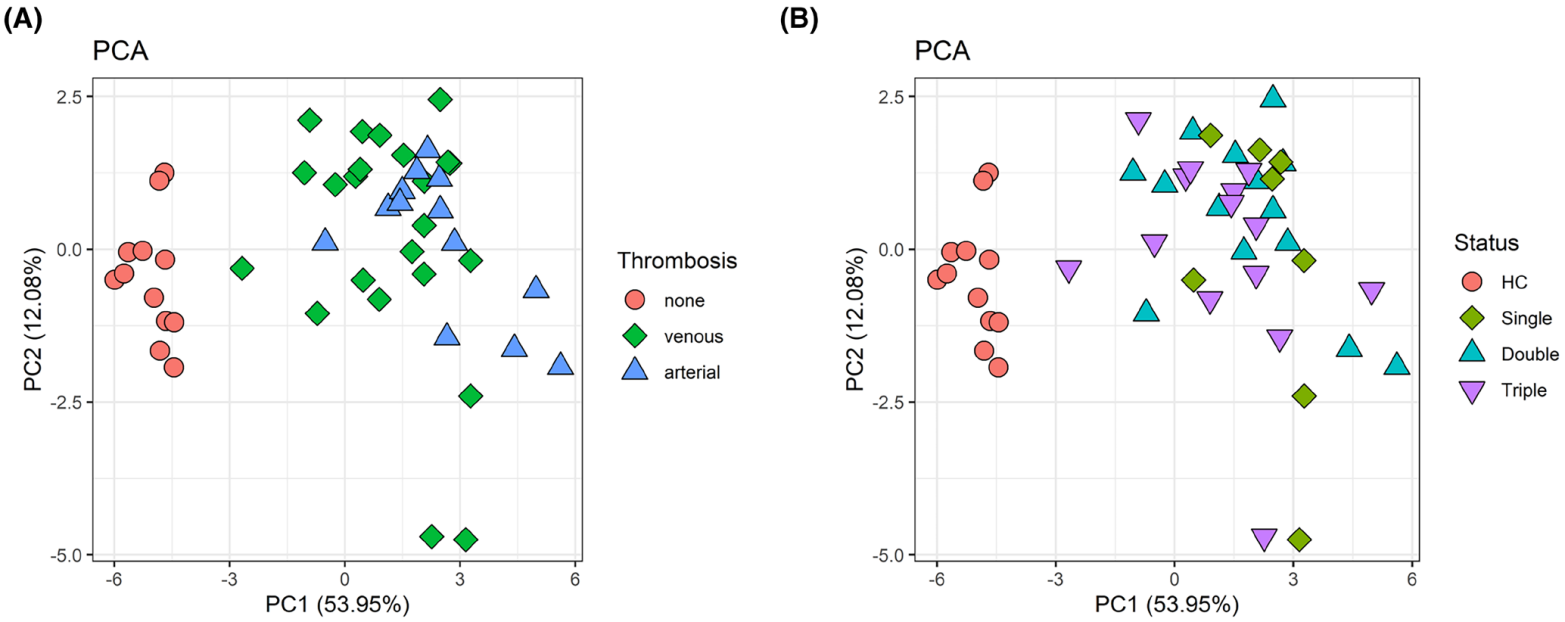
Principal component analysis (PCA). PCA was performed on transformed and normalised data. (A) PCA colour and shape depending on type of thrombosis (None, HC; venous thrombosis or arterial thrombosis). (B) PCA colour and shape depending on aPL status (HC, or single, double or triple positive aPL.

### Plasma cytokine titres are higher in patients with arterial thrombosis compared to venous

Hierarchical clustering revealed that a stronger cytokine signature may be associated with arterial thrombosis compared to venous thrombosis. We therefore compared the concentration of plasma cytokines between venous and arterial thrombosis (Figure 5). Cytokine titres were significantly higher in samples from arterial thrombosis compared to venous thrombosis for GM‐CSF, IFN‐α, IL‐6, IL‐10, IL1α, IL‐4, IL‐17a and TNF‐α. While not statistically significant, there was a trend towards higher ICAM‐1 levels in patients with venous versus arterial thrombosis, indicating operation of different thrombotic pathways. No significant differences were found between groups for IL‐1β, IFN‐γ, IL‐8, IL‐12p70, IL‐13, IP‐10, MCP‐1, MIP1α, MIP1β, P‐selectin or E‐selectin (Figure S5).

**FIGURE 5:**
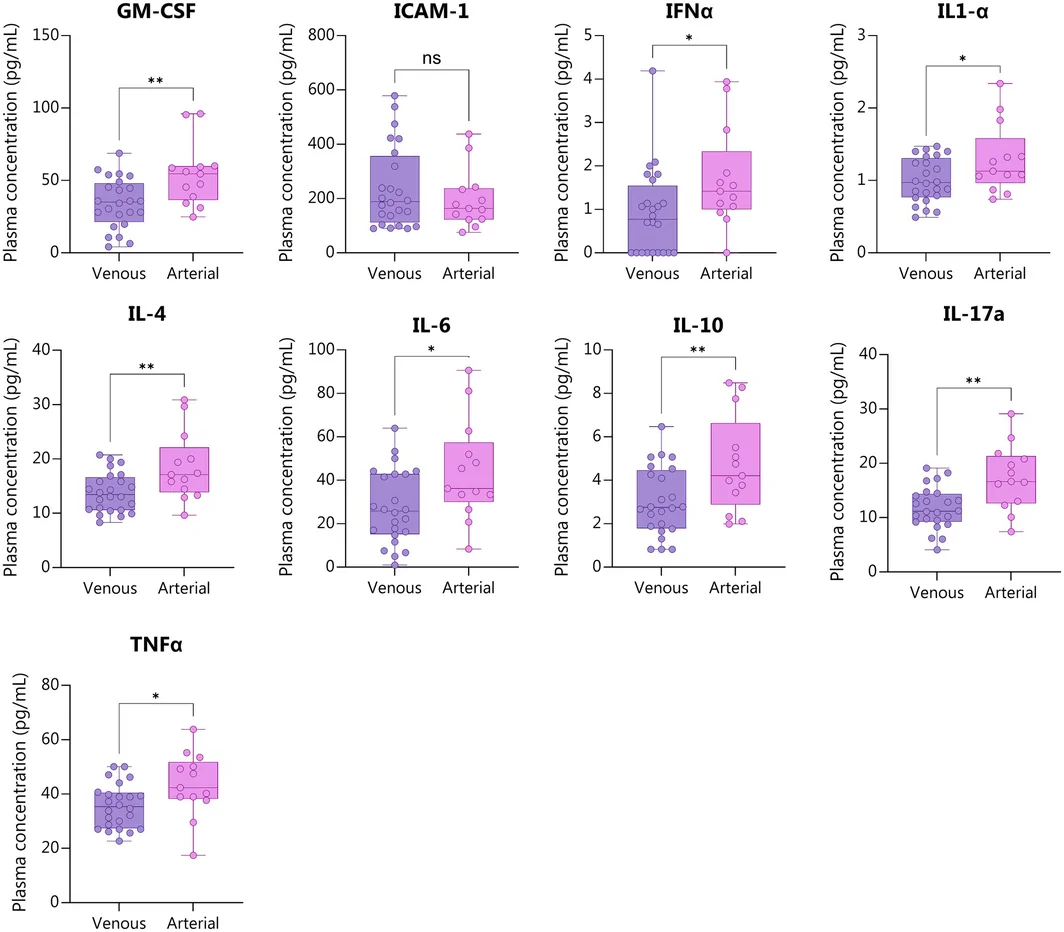
Cytokine titres differ between APS patients experiencing venous and arterial thrombosis. Cytokines measured by MultiPlex assay were compared between APS patients that experienced venous thrombosis (*n* = 24) and arterial thrombosis (*n* = 13). Box plots show the median, interquartile range, along with minimum and maximum values. Statistical significance was tested for using an unpaired *t* test. *p < 0.05, **p < 0.01, ***p < 0.005, ****p < 0.0001.

## DISCUSSION

In this study, we demonstrate that patients with thrombotic APS exhibit a broad pro‐inflammatory cytokine signature compared with HC despite stable anticoagulation with warfarin for at least 3 months following the thrombotic event. Importantly, our data suggest that arterial thrombosis is associated with a more pronounced inflammatory profile than venous thrombosis, supporting the concept that despite similar exposure to aPL, divergent inflammatory pathways may contribute to different thrombotic manifestations.

The cytokine analysis identified significantly elevated levels of multiple inflammatory mediators in thrombotic APS, including GM‐CSF, IFN‐γ, IL‐1α, IL‐1β, IL‐6, IL‐17a and TNF‐α, alongside adhesion and endothelial activation markers such as ICAM‐1 and P‐selectin. These findings are consistent with growing evidence that APS is not only a prothrombotic disorder, but also an inflammatory condition characterised by sustained innate and adaptive immune activation.^13^ Statistical significance for individual cytokines was determined using nominal *p* values without multiple testing correction. Given the exploratory design, limited sample size, and highly correlated cytokine measurements, adjustment could substantially increase false‐negative findings. These analyses were intended to identify candidate biomarkers for future study rather than establish definitive associations, while acknowledging an increased risk of type I error.

The elevation of IL‐1 family cytokines and TNF‐α is particularly noteworthy given their established role in vascular inflammation and thrombosis.^14^ IL‐1β and TNF‐α both promote endothelial activation and tissue factor expression.^15^ Similarly, IL‐6 has recognised prothrombotic effects through induction of acute phase responses and enhancement of platelet activation.^16^ Increased IL‐17a levels may reflect Th17 pathway activation,^17^ which has been implicated in APS pathogenesis and vascular injury.^18^ The concurrent increase in anti‐inflammatory cytokines such as IL‐10 may represent compensatory immune regulation in response to ongoing inflammation. Together, these findings suggest that thrombotic APS is characterised by a complex inflammatory milieu involving both innate and adaptive immune pathways.

A study comprising 101 patients with thrombotic primary APS showed 8.6‐fold increased levels of TNF‐α and 66% increased levels of tissue factor compared to controls (*n* = 131).^6^ Furthermore, TNF‐α and IL‐8 levels were higher in APS patients with venous thrombosis, compared to patients with arterial thrombosis and controls.^6^ This higher TNF‐α level in arterial thrombosis compared to venous thrombosis, which is not seen in our study, may reflect the lower number of patients with additional vascular risk factors in our study cohort (Table 1) compared to the cited study. In another study of 250 APS patients and 129 HC, significantly increased concentrations of IL‐2, IL‐1β, IL‐12p70, IL‐8, IL‐6 and IFN‐γ, and reduced IL‐5, IL‐17a and TNF‐α were reported.^7^ The study further suggested that patients with thrombotic APS have significantly higher concentrations of IL‐6, IL‐10 and TNF‐α compared to obstetric APS patients.^7^ These studies collectively show a trend towards elevated pro‐inflammatory cytokines (notably IL‐6, TNF‐α, IL‐1β, IL‐8 and adhesion molecules) in patients with APS, particularly those with thrombotic manifestations, aligning well with our findings. They underscore the inflammatory contribution to thrombosis in APS beyond traditional coagulation pathways.

An important finding of this study is the identification of distinct cytokine‐defined patient clusters within thrombotic APS. Hierarchical clustering separated patients into three principal inflammatory groups distinct from HC. Although several cytokines, including GM‐CSF, IL‐6, IL‐10, IL‐12p70, IL‐13 and TNF‐α, were consistently elevated across all thrombotic APS clusters, other cytokines demonstrated cluster‐specific expression patterns. In particular, cluster 3 exhibited a stronger inflammatory signature characterised by elevated IFN‐α expression. This observation is biologically plausible, as type I IFN signalling has been recognised as an important pathway in APS.^19^ Transcriptomics studies in APS, including bulk RNA (ribonucleic acid) sequencing or microarray analysis of aortic valve tissue, whole blood, neutrophils and human umbilical vein endothelial cells treated with APS patient‐IgG demonstrated IFN‐regulated genes and signalling are upregulated in APS.^20^, ^21^, ^22^, ^23^, ^24^, ^25^ In a study that used an unbiased plasma proteomic screen in 40 persistently aPL‐positive patients (aPL carriers, thrombotic APS, microvascular APS and obstetric APS, *n* = 10 each category), a shared thrombo‐inflammatory signature was identified involving coagulation, complement and immune activation pathways, increasing with APS severity.^26^

Interestingly, in our study, we identified different clusters of patients based on thrombotic phenotype. Cluster 3 was enriched for patients with arterial thrombosis, whereas clusters 2 and 4 predominantly represented venous thrombosis. Furthermore, direct comparison between arterial and venous thrombosis demonstrated significantly higher cytokine levels in arterial disease for GM‐CSF, IFN‐α, IL‐6, IL‐10, IL‐1α, IL‐4, IL‐13, IL‐17a and TNF‐α. These findings suggest that arterial thrombosis in APS may represent a more inflammatory vascular phenotype than venous thrombosis. This is consistent with clinical observations that arterial APS frequently exhibits greater association with endothelial dysfunction, platelet activation and accelerated atherosclerosis‐like pathology compared with venous disease, which may be more dependent upon coagulation activation and venous stasis.^27^ Of note, in our study there was no difference in the proportion of patients with dyslipidaemia in venous versus arterial thrombosis.

Despite the inflammatory differences observed between thrombotic phenotypes, aPL status itself did not associate with plasma cytokine levels on PCA analysis. This finding is somewhat unexpected given the established pathogenic role of aPL antibodies in APS. However, it is possible that once thrombosis has occurred, downstream inflammatory responses become partially dissociated from aPL titre or profile. The fact that ≥75% of the patients with triple positive aPL were on HCQ compared to none of the patients with single or dual positive aPL may also contribute to this effect and the observed lack of difference between patients on HCQ versus no HCQ. In a study of 27 patients with PAPS, of whom 37% were triple positive for aPL, treatment with HCQ for 6 months was associated with a significant reduction in cytotoxic T lymphocytes, low‐density lipoprotein (LDL) cholesterol and serum glucose levels, together with a significant increase in regulatory T lymphocytes compared with baseline values.^28^ HCQ use only in triple positive patients may therefore mask group differences. However, the lack of association between cytokine profile and demographic factors such as age, sex and BMI strengthens the interpretation that the observed inflammatory differences are disease‐related rather than confounded by baseline clinical variables.

The trend towards higher levels of ICAM‐1 in venous compared with arterial thrombosis may suggest differing mechanisms of vascular activation between thrombotic phenotypes. ICAM‐1 is closely linked to endothelial‐leucocyte interactions and venous endothelial activation.^29^ It is plausible that venous thrombosis in APS is characterised by relatively greater endothelial adhesion pathway activation, whereas arterial disease is driven more strongly by systemic inflammatory cytokine networks and platelet‐mediated inflammation.

In an APS mouse model, ciprofloxacin reduced pregnancy loss and disease manifestations compared with ceftazidime^30^ in association with increased IL‐3 messenger ribonucleic acid (mRNA) expression and protein production, and upregulation of GM‐CSF.^30^ In vitro, APS IgG impaired trophoblast invasiveness, differentiation and human chorionic gonadotropin secretion by binding to trophoblast cells, and treatment with IL‐3 restored these functions.^31^ In contrast to these studies of experimental models, obstetric APS and selected inflammatory mediators, the current study includes comprehensive profiling of circulating cytokines in well‐characterised thrombotic APS cohorts, providing insight into the inflammatory mechanisms underlying thrombosis and highlighting potential biomarkers and therapeutic targets.

Collectively these findings support thrombotic APS cytokine signature linkage to persistent thrombosis risk and potential immunomodulatory targets explaining recurrent thrombosis occurrence in 30%–46% of patients despite anticoagulation.^32^, ^33^ In addition to providing further information supporting APS as a thrombo‐inflammatory disease, the cytokine concentrations defined in this study may serve as a useful reference framework for researchers developing in vitro, in vivo, or computational models of thrombotic APS, facilitating physiologically relevant modelling of the inflammatory environment accompanying APS‐related thrombosis.

Our study has several limitations. First, the small sample size, particularly within arterial and venous subgroup analyses. Larger studies will be required to validate these findings and determine whether distinct biomarker profiles can reliably discriminate arterial from venous APS. Second, this was a cross‐sectional study, and cytokine levels may vary over time. Longitudinal studies are needed to determine whether these cytokine profiles remain stable and whether they predict recurrent thrombosis or clinical outcomes. Third, some patients were receiving HCQ, which has immunomodulatory effects and may influence circulating cytokine levels. However, there was no difference in the number of patients receiving HCQ in venous versus arterial thrombosis. Mechanistic studies are required to determine whether these cytokines directly contribute to thrombosis or simply reflect downstream immune activation. It is important to note that while differences in cytokine levels are identified between venous and arterial thrombosis, these samples incompletely separate in the PCA space. Factor(s) that were not identified in this study may more robustly separate samples. For instance, it is widely accepted that a ‘second hit’ may induce thrombosis in individuals with persistently positive aPL. The variable nature of such second hits may well explain differences in cytokine titres and thrombotic type. The absence of a thrombotic control group without APS limits our ability to determine whether the cytokine elevations are specific to APS or reflect prior thrombosis more generally. Additionally, we included only patients with a single thrombotic event, and the inflammatory pattern may be different in APS with recurrence despite stable anticoagulation.

Despite limitations, our findings support significant inflammatory dysregulation and distinct APS inflammatory subgroups. The inflammatory signatures identified in this study may have translational impact in thrombotic APS by improving patient stratification complementing conventional clinical and aPL‐based assessment. Distinct cytokine profiles could identify inflammatory endotypes associated with thrombotic risk, monitor disease activity and assess therapeutic response. Dysregulated cytokine networks also highlight immune pathways as targets for personalised immunomodulatory therapies, particularly in patients with recurrent arterial thrombosis. However, these findings remain exploratory and require prospective validation in larger, independent cohorts before their predictive value, reproducibility and clinical utility can be established.

In conclusion, thrombotic APS is characterised by broad upregulation of pro‐inflammatory cytokines and heterogeneous inflammatory phenotypes despite stable anticoagulation. Distinct cytokine clusters were identified, with arterial thrombosis associated with a stronger inflammatory signature than venous thrombosis. These findings support the concept that thrombotic APS comprises biologically diverse subgroups that inflammatory pathways may differentially contribute to arterial and venous thrombosis, implying a tailored therapeutic approach may improve patient outcomes in the future. The identification of an IFN‐α‐enriched inflammatory phenotype associated with arterial thrombosis provides a rationale for biomarker‐guided risk stratification and targeted immunomodulatory approaches in APS. However, the small number of patients within each cluster warrants cautious interpretation of these exploratory findings. Validation in larger, independent cohorts with diverse clinical phenotypes is required before establishing their biological or clinical relevance. Future studies integrating cytokine profiling with longitudinal outcomes, additional molecular biomarkers and thrombotic non‐APS controls will be essential to determine the reproducibility and translational utility of these inflammatory signatures in thrombotic APS.

## AUTHOR CONTRIBUTIONS

**Deepa J. Arachchillage:** Conceptualization; funding acquisition; writing – original draft; writing – review and editing; supervision; resources; project administration; visualization; validation; methodology. **Charis Pericleous:** Writing – review and editing. **Megan V. Preece:** Writing – review and editing. **Mike Laffan:** Writing – review and editing. **Simon Abrams:** Investigation; writing – review and editing. **Catherine B. Ducker:** Writing – original draft; formal analysis. **Jeremy Schofield:** Investigation; writing – review and editing.

## FUNDING INFORMATION

Deepa J. Arachchillage is funded by Medical Research Council UK (MR/Z505274/1) and infrastructure support for some of the work and funding for Megan V. Preece was provided by the NIHR Imperial Biomedical Research Centre (BRC).

## CONFLICT OF INTEREST STATEMENT

The authors have no conflicts of interest to disclose.

## Supporting information


**Figure S1.** Cytokine plasma concentrations unchanged from healthy controls.
**Figure S2.** Principal component analysis (PCA) shows no apparent association between clinical characteristics and variation in the data.
**Figure S3.** Principal component analysis (PCA) shows no apparent association between specified variables and variation in the data.
**Figure S4.** Cytokine titres do not significantly differ between APS patients on hydroxychloroquine versus no hydroxychloroquine except for MIP1β.
**Figure S5.** Cytokine titres that do not significantly differ between APS patients experiencing venous and arterial thrombosis.

## Data Availability

The data that support the findings of this study are available on request from the corresponding author. The data are not publicly available due to privacy or ethical restrictions.
