# Supplementary material for: A pro‐inflammatory cytokine signature characterises thrombotic antiphospholipid syndrome despite stable anticoagulation
Source: Br J Haematol. 2026 Sep 9;209(5):2233–43. doi: 10.1111/bjh.70816 (PMC13748370; doi:10.1111/bjh.70816)
Supplement: Supplementary file 1 [file BJH-209-2233-s001.docx]

**Supplementary Figure 1: Cytokine plasma concentrations unchanged from healthy controls.** Cytokine concentrations were measured in plasma of thrombotic APS patients (n=38) and healthy controls (HC, n=12) by Multiplex assay. The median is identified with a thick black line, and quartiles with thin black lines. Statistical significance was tested for using an unpaired t test with Welch’s correction. ns = p>0.05.

**Supplementary Figure 2: Principal Component Analysis (PCA) shows no apparent association between clinical characteristics and variation in the data.** PCA was performed on transformed and normalised data. (A) coloured by biological sex, (B) by age and (C) by BMI.

**Supplementary Figure 3:** **Principal Component Analysis (PCA) shows no apparent association between specified variables and variation in the data.** PCA was performed on transformed and normalised data. N/A indicates categorical variables not applicable to healthy controls. (A) coloured by use of HCQ, (B) by categorised titres of αβ2GPI (IgM or IgG), (C) by categorised titres of αCL, (D) by presence of lupus anticoagulant (LA), (E) by categorised titres of complement C3, (F) by categorised titres of complement C4, and (G) by sufficiency (50+) or insufficiency (<50) of vitamin D.

**Supplementary Figure 4:** Cytokine titres do not significantly differ between APS patients on hydroxychloroquine vs no hydroxychloroquine except for MIP1β. Cytokine concentrations were measured in the plasma of thrombotic APS patients by Multiplex assay. The plasma concentration for each cytokine is shown for those not taking HCQ (n=26) and those on HCQ (n=11). The median is identified with the thick black line. Statistical significance was tested for using an unpaired t test. * p<0.05.

**Supplementary Figure 5: Cytokine titres that do not significantly differ between APS patients experiencing venous and arterial thrombosis.** Cytokines measured by MultiPlex assay were compared between APS patients that experienced venous thrombosis (n=24) and arterial thrombosis (n=13). Box plots show the median, interquartile range, along with minimum and maximum values. Statistical significance was tested for using an unpaired t test. ns = p>0.05.
